# The Connection Between Stress and Women’s Smoking During the Perinatal Period: A Systematic Review

**DOI:** 10.3390/brainsci15010013

**Published:** 2024-12-26

**Authors:** M. Carmen Míguez, Yara Queiro, Cristina M. Posse, Alba Val

**Affiliations:** Department of Clinical Psychology and Psychobiology, Faculty of Psychology, Institute of Psychology (IPsiUS), University of Santiago de Compostela, Campus Vida, 15782 Santiago de Compostela, Spain; yara.queiro@rai.usc.es (Y.Q.); cristinamaria.posse.cepeda@usc.es (C.M.P.);

**Keywords:** psychological stress, smoking, women, pregnancy, postpartum period

## Abstract

Introduction. In women, smoking during pregnancy and the postpartum period has important consequences for maternal and infant health, and interventions to assist smoking cessation during this period are essential. Although smoking has been associated with the presence of mental health problems, few studies addressing the factors associated with perinatal smoking have examined the role of stress. The aim of this review was to identify the relationships between the presence of stress and smoking during pregnancy and the postpartum period. Method. A literature search of the PsycInfo, Pubmed and Web of Science databases was carried out to identify relevant articles published between January 2013 and June 2024. A total of 15 studies that met the inclusion criteria were selected for review. Results. Twelve of the studies analysed the relationship between stress and smoking during pregnancy, two studies involved the postpartum period, and one study included both periods. Diverse instruments were used to assess stress, although the PSS-14 was the most commonly used. Eleven of the studies found a relationship between stress and smoking in the perinatal period, with stress being a predictor of smoking. Conclusions. The findings highlight the need to consider stress management when developing effective interventions to help women quit smoking during pregnancy and maintain abstinence in the postpartum period.

## 1. Introduction

Smoking is a public health problem that causes more than 8 million deaths annually worldwide [1]. Most of these deaths are due to diseases, such as cancer, chronic obstructive pulmonary disease and cardiovascular disease [2]. In women, smoking can lead to dysmenorrhoea, early menopause, osteoporosis and an increased likelihood of certain cancers, such as cervical and breast cancer [2,3]. Smoking during pregnancy can have consequences for both the pregnant woman and the foetus. Tobacco consumption (or exposure to tobacco smoke) during pregnancy is the leading preventable cause of miscarriage and neonatal death. It also increases the likelihood of pre-eclampsia, ectopic pregnancy, placental abruption or premature rupture of the amniotic membrane. In addition, it can lead to birth defects and/or low birth weight. Some other detrimental effects extend into childhood, such as an increased risk of behavioural problems and neurodevelopmental disorders [2,4].

The prevalence of tobacco consumption during pregnancy remains high despite an awareness of the consequences. A recent review study found that 21% of pregnant women smoke [5], and similar rates have also been reported in other studies, i.e., 15.7% [6] and 19.7% [7]. A meta-analysis reported that 53% of women who smoked daily continued to do so during pregnancy [8]. The same study also found that the prevalence of gestational smoking across the world was highest in Ireland (38.4%), followed by Uruguay (30%), Bulgaria (29.4%), Spain (26%) and Denmark (25%).

Moreover, the percentage of women who spontaneously quit smoking is higher in pregnant women than in women in the general population. Between 43.6% [6] and 56.1% [9] of women quit smoking during pregnancy.

Women who have smoked throughout their pregnancy are joined by those who relapse during the postpartum period after having quit during pregnancy. Therefore, smoking prevalence is often higher in the postpartum period than during pregnancy. Relapse rates ranging from 15.2% at 2 months postpartum [10] to 42.6% at 6 months postpartum [11] have been reported.

Psychological factors, such as stress, have been associated with an increased likelihood of smoking because smokers may use tobacco as a coping strategy [12,13]. On the other hand, research considering gender differences [14] indicates that the association between stress and smoking is stronger in women, who are more likely than men to use tobacco to relieve stress.

Pregnant women with low levels of depression, anxiety and/or perceived stress seem to be able to quit smoking more easily [15]. Likewise, higher levels of anxiety, depression and perceived stress have been associated with a greater likelihood of relapse in the postpartum period [10,15].

Mental health problems, including stress, during the perinatal period also have consequences for maternal and child health. Exposure to high levels of stress during pregnancy has been linked to an increased likelihood of developing pre-eclampsia, gestational diabetes and even delayed breast milk production [16]. Stress also has psychological consequences in women during pregnancy, as it is considered a precursor of postpartum depression or worsening of mental disorders that are already present [17]. Regarding the consequences that stress during pregnancy can have on offspring, it was found [18] that children whose mothers reported having experienced stress during pregnancy were more likely to experience psychiatric disorders in the future, especially mood disorders. Other research has indicated a correlation between stress during pregnancy and neurodevelopmental complications in children, leading to cognitive and motor problems as well as behavioural and anxiety disorders [19]. Some studies indicate that stress during pregnancy may also increase the likelihood of children suffering from asthma, growth problems and allergies [20,21].

In addition to the consequences that stress and smoking each generate during the perinatal period, these variables can act together, with various consequences for child health. Children of mothers who had smoked and been stressed during pregnancy had a difficult temperament, lower levels of executive control and more disruptive behaviour [22]. Stress and passive exposure to tobacco smoke during pregnancy also increase the likelihood of children suffering from internalising disorders in early childhood [23].

Research on the psychological variables related to smoking during pregnancy and the postpartum period has mainly focused on the role of anxiety and depression [24,25]. Although stress and smoking are known to be linked, evidence regarding how the two variables are related is scarce, especially in pregnant women. The aim of this review was, therefore, to determine how stress and smoking are related during pregnancy and the postpartum period. The specific objectives were as follows: (1) to identify the instruments used to assess stress during pregnancy and postpartum; (2) to determine whether women who smoke during pregnancy experience higher levels of stress than non-smokers; (3) to determine whether women who quit smoking during pregnancy and relapse in the postpartum period experience higher levels of stress; (4) to determine whether stress is a cause or a consequence of smoking in women during pregnancy and the postpartum period. If a positive and significant relationship is found between stress and smoking during the perinatal period, future interventions should include addressing stress to support smoking cessation during pregnancy and maintenance of abstinence in the postpartum period.

## 2. Method

This study’s selection process was carried out following the recommendations of the Preferred Reporting Items for Systematic reviews and Meta-Analyses (PRISMA) guidelines [26]. Thus, the articles had to meet the following inclusion criteria: (1) be published in a scientific journal; (2) written in Spanish or English but conducted in any country; (3) be an empirical study; (4) clearly define what is meant by stress and how it has been evaluated; (5) clearly define what is meant by being a smoker or non-smoker; (6) analyse the relationship between smoking and stress in women during the perinatal period (pregnancy and the first postpartum year); (7) the sample is made up of pregnant or postpartum women over 18 years of age, smokers or ex-smokers; and (8) published between January 2013 and June 2024.

### 2.1. Search Strategy

A literature search of the PsycINFO, Pubmed and Web of Science (WOS) databases was carried out to identify papers published between the specified period (1 January 2013 to 31 June 2024). The keywords used in the initial search were combined as follows: “stress” (abstract/title) AND “smok*” OR “tobacco” (abstract/title) AND “pregnan*” OR “antenatal” OR “prenatal” OR “perinatal” OR “postnatal” OR “postpartum” (abstract/title) AND “women” OR “woman” (abstract/title).

### 2.2. Strategy for the Selection of Studies and Analysis of Results

**Figure 1:**
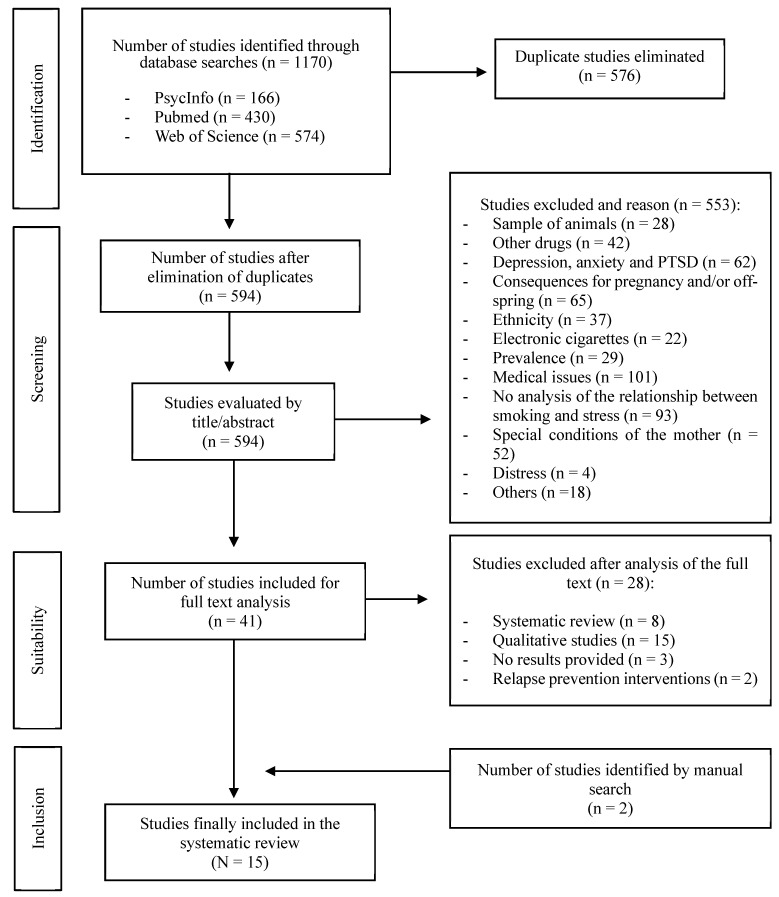
PRISMA diagram outlining the article selection process.

The selection process was carried out independently by two researchers, and in the case of any discrepancies, a third author decided whether the article should be included. All publications that met the search criteria were transferred to Refworks, and all repeated publications were removed. The researchers read the title and abstracts of the initially selected articles and then read the full text of the articles that passed this stage of the process. The PRISMA flow diagram in Figure 1 summarises the study selection process.

For better understanding of the results reported, the studies were divided into two categories. The first category included studies that examined the relationship between stress and smoking and/or smoking cessation during pregnancy. The second category grouped studies that examined the relationship between stress and postpartum smoking relapse. The extracted data were categorised by year, objective, sample, measures, results and conclusions. Statistical data from the studies were evaluated.

## 3. Results

### 3.1. Study Selection

A total of 1170 potential scientific publications were identified in the initial search strategy: PsycINFO (*n* = 166), Pubmed (*n* = 430) and WOS (*n* = 574). Of these, 576 were duplicated, and the number of publications remaining after elimination of duplicates was 594.

A systematic search was then carried out by screening the retrieved studies. A series of inclusion and exclusion criteria were used to guide the selection process. After reading the title and abstract of the studies, the researchers excluded a total of 553 studies because they (1) were conducted on animal samples, (2) involved other drugs (i.e., cannabis, cocaine, opioids), (3) considered depression, anxiety or post-traumatic stress disorder during the perinatal period, (4) focused on the consequences of tobacco consumption for pregnancy and/or offspring, (5) focused on analysing the influence of ethnicity on tobacco consumption, (6) analysed the use of electronic cigarettes, (7) focused on analysing smoking prevalence (overall or in different countries), (8) analysed medical issues (e.g., hormones, physical diseases, etc.), (9) did not consider the relationship between smoking and stress (but analysed variables other than stress), (10) tested samples from pregnant women or mothers with special conditions (e.g., HIV), (11) examined the relationship between tobacco consumption and distress during the perinatal period (this variable was discarded after a general review, and it was noted that it is a term that is not yet well defined and for which there are no valid and reliable assessment instruments) and (12) were studies that did not fall into any of the previously discussed categories.

The researchers then read the full text of each of the 41 articles selected and finally included 13 studies; the other 28 studies were excluded because they were systematic reviews (*n* = 8), were qualitative studies (*n* = 15), did not provide results (*n* = 3) or were interventions focused on relapse prevention (*n* = 2). A further two studies, identified by a manual search during the reading of the studies included in the review, were added. Therefore, 15 articles were finally included in the review.

The quality of the selected studies was assessed using the Mixed Methods Assessment Tool [27] through two general screening questions and five specific questions, depending on the design of each study (in this case, descriptive quantitative trials) (Table S1). The researchers re-read the full text of each article, extracting the information of interest/relevance for the review. The selected results were then compiled in table format.

### 3.2. Characteristics of Selected Studies

Fifteen articles examining the relationship between stress and smoking during pregnancy and postpartum were reviewed. Of these, 12 studies examined the relationship between the two variables only during pregnancy, two studies considered only the postpartum period, and one study considered both periods.

The 15 studies were carried out in different countries. Most (*n* = 9) were conducted in the USA [28,29,30,31,32,33,34,35,36]. Two were conducted in the Netherlands [37,38], and the others were conducted in Spain [6], Canada [39], Lithuania [40] and Brazil [41].

### 3.3. Instruments Used to Assess Stress in the Perinatal Period

The instruments used in the reviewed studies are described below to determine which are the most commonly used to assess stress in the perinatal period (see Table 1). The instruments are grouped into two sections: general instruments that assess stress and specific instruments that assess perinatal stress.

#### 3.3.1. General Instruments Assessing Stress

Among the studies selected (*n* = 15), nine used general instruments to assess stress in pregnancy and the postpartum period: the PSS-14 (*n* = 6), the LEI (*n* = 1) and ad hoc questions (*n* = 2).

The PSS-14 (*Perceived Stress Scale* [42]) is a self-report instrument used to assess the level of perceived stress during the past month. It consists of 14 items with a 5-point Likert-type response format referring to the frequency with which the person experiences stress symptoms, with higher scores indicating higher levels of perceived stress. Scores of 0–14 indicate that the respondent is never or hardly ever stressed; scores of 15–28 indicate that the respondent is occasionally stressed; scores of 29–42 indicate that the respondent is often stressed; and scores of 43–56 indicate that the respondent is very often stressed. The PSS-14 is one of the instruments most widely used to assess stress, and it was used in six of the studies reviewed [6,29,30,31,33,41].

*The LEI* (*Life Events Inventory* [43]) consists of a checklist of possible stressful life events (domestic, personal and work) that participants are asked to complete by ticking off whether they have experienced any of the 55 events in the last 6 months. It was used by one study [28] that adapted the instrument by applying only 13 items.

Ad hoc *question on the stress suffered during pregnancy.* In one study [39], the level of stress experienced before or during pregnancy by the mothers participating in the research was assessed by the following question: ‘Thinking about the amount of stress in your life during the 12 months before the birth of your baby, would you say that most days were “not at all stressful”, “somewhat stressful” or “very stressful”?’.

Ad hoc *questionnaire on stressful or traumatic experiences*. Another study [40] administered a questionnaire that included a question about whether participants had experienced any stressful or traumatic experiences during pregnancy.

#### 3.3.2. Specific Instruments Assessing Perinatal Stress

Five of the 15 studies reviewed used one of the following specific instruments to assess stress during the perinatal period: the NuPDQ (*n* = 1), the CWS (*n* = 1), the ESI (*n* = 1), the PRAMS-SLE (*n* = 2) or the Events Questionnaire (*n* = 1).

*The NuPDQ* (*Revised Prenatal Distress Questionnaire* [44]) consists of 17 items that assess the intensity with which the pregnant woman has experienced symptoms of pregnancy-related stress. It is particularly appropriate for assessing pregnancy-specific stress. The items assess concerns about the baby’s health, body changes, the respondent’s ability to care for the baby and maternal health problems. Responses are distributed on a Likert-type scale that rates how worried the respondents feel in regard to each item, with higher scores indicating higher levels of stress. This questionnaire was used by one study [38].

*The CWS* (*Cambridge Worry Scale* [45]) is a 17-item questionnaire that measures the severity of the worries most experienced by women during pregnancy. Scores are rated on a Likert-type scale ranging from 0 (‘not a worry’) to 5 (‘a major worry’), with higher scores indicating more serious worries. The questionnaire includes four subscales (socio-medical, self-health, socio-economic and interpersonal relationships). This instrument was used in one study [38].

*The ESI* (*Everyday Stressors Index* [46]) is a self-report instrument that assesses common problems faced daily by low-income mothers with young children. It consists of 20 items, which are rated on a 4-point Likert-type scale and classified according to five types of problems: economic worries, role overload, work problems, parenting problems and interpersonal conflicts. The total score ranges from 0 to 60, with higher scores indicating higher levels of worry about stressful daily problems. Of the 15 studies reviewed, it was used in one study [35].

*The Pregnancy Risk Assessment Monitoring System Survey Stressful Life Events (PRAMS SLE* [47]). This survey aims to identify and track selected maternal experiences before, during and after pregnancy. The PRAMS SLE is based on the Modified Life Events Inventory [48]. The latest version of the scale includes 26 items on different types of stressful life events, and participants are asked whether or not they have experienced these events. The following are examples of items: ‘I had problems conceiving a baby’, ‘I experienced a chronic illness related to my pregnancy’, ‘I was worried about my return to work after childbirth’. This survey was used in two studies [32,36].

*Event questionnaire* [49] consists of 47 stressful life events classified in eight categories. Participants respond regarding whether they have experienced any of these events during pregnancy by indicating their perceived severity on a Likert-type scale. One study [37] used this questionnaire to assess stress.

In summary, the PRAMS SLE was used by two of the five studies, and each of the remaining instruments was used in one study.

Thus, nine different instruments were used to assess stress in the 15 studies reviewed. Four assessed general stress (PSS-14, LEI, one question and one ad hoc questionnaire), and five assessed specific stress in the perinatal period (NUPDQ, CWS, ESI, PRAMS-SLE and Event Questionnaire). The instrument most commonly used to assess stress in the perinatal period was Cohen’s PSS-14 [42], which was used in six studies [6,29,30,31,33,41].

### 3.4. Relationship Between Stress and Tobacco Consumption

The findings of the selected studies on the relationship between stress and smoking during pregnancy are presented below to examine whether women who smoke have higher levels of stress. The studies assessing the relationship between stress and smoking and/or smoking cessation during pregnancy are reported first, followed by the results of the studies relating stress to postpartum smoking relapse.

#### 3.4.1. Relationship Between Stress and Smoking and/or Smoking Cessation During Pregnancy

Of the 15 studies reviewed, 13 examined the relationship between stress and smoking or quitting during pregnancy (see Table 2). Specifically, the relationship between stress and tobacco consumption was examined in ten studies [6,28,31,33,35,36,37,38,40,41]; the relationship between stress and quitting smoking was addressed in two studies [34,39]; and the relationship between stress, tobacco consumption and smoking cessation was examined in one study [30].

Seven of the 10 studies that examined the relationship between stress and smoking during pregnancy reported a statistically significant relationship [6,28,31,33,35,36,41]. Thus, women with higher levels of stress during pregnancy are more likely to continue smoking during pregnancy.

Similarly, the two studies that examined spontaneous smoking cessation during pregnancy [34,39] found that pregnant women who experienced lower levels of stress were more likely to quit smoking.

The only study that considered both smoking and smoking cessation during pregnancy [30] also reported a statistically significant association between being stressed and smoking and not being stressed and quitting smoking.

#### 3.4.2. Relationship Between Stress and Postpartum Smoking Relapse

All three studies addressing the relationship between stress and postpartum relapse [28,32,39] found a statistically significant relationship between the two variables (see Table 3). Only one study [29] reported the timing of the assessment, which was at eight weeks postpartum.

### 3.5. Stress as a Cause or Consequence of Smoking in Pregnancy and the Postpartum Period

Considering the results of the reviewed studies, regarding the direction of the relationship between stress and smoking during pregnancy and postpartum, allowed for us to categorise the studies into two groups depending on whether stress is the independent or dependent variable.

*Stress as an independent variable.* Of the 15 studies reviewed, 13 found stress to be the main variable that predicts or determines smoking in the perinatal period [6,28,29,30,32,34,35,36,37,38,39,40,41]. Ten studies found a significant relationship between stress and smoking during pregnancy [6,28,30,34,35,36,39,41] and between stress and postpartum relapse [28,32,39]. Therefore, these studies concluded that stress would be a cause of women smoking during pregnancy and relapse postpartum.

*Stress as a dependent variable.* Two of the 15 selected studies found that smoking can lead to women experiencing increased stress [31,33]. Both found a significant relationship between smoking and stress during pregnancy. According to these studies, tobacco consumption during pregnancy increases the likelihood of higher levels of stress in the expectant mother [33] and leads to a greater sense of lack of self-control [31].

## 4. Discussion

The main objective of this review was to identify whether there is a relationship between stress and smoking during pregnancy and the postpartum period and to clarify the direction of any relationship in pregnant women.

The first objective was to determine which instruments are most commonly used to assess stress in the perinatal period. Although there is no consensus among researchers as to which instrument is the most appropriate, as nine different instruments are used, the PSS-14 [42] was the most widely used. This is also one of the most widely used tools to assess stress in the general population. The diversity of the instruments may be due to the different definitions of stress depending on the approach used in the research. For example, the cognitive approach focuses on what the person thinks about the stressful stimulus, whereas the behavioural approach focuses on what the person does in response to the stressful stimulus. On the other hand, some studies assessed general stress symptoms [6,28,29,30,31,33,39,40,41] and other pregnancy-specific stress symptoms [32,35,36,37,38], and therefore, the selection of instruments differed according to the type of stress being assessed. Moreover, stress can be assessed by different parameters, such as the presence or absence of stress [40], the level of stress [6,29,30,39,41] and the number or severity of stressful life events [28,32,35,36,37]. Therefore, the tool used to assess stress will depend on the aims of each study.

As a second objective, we tried to determine whether women who smoke during pregnancy are more stressed than non-smokers, which would indicate a relationship between stress and smoking during pregnancy. Of the 15 studies selected, 13 examined the relationship between stress and smoking and/or smoking cessation during pregnancy. Most of these studies (*n* = 11) reported a significant relationship between stress and tobacco consumption [6,28,30,31,33,35,36,38,41] and between stress and smoking cessation during pregnancy [30,34,39]. It was found that women who smoke during pregnancy score higher on stress than those who do not smoke or those who quit smoking when they found out they were pregnant. Similarly, those who stopped smoking reported lower levels of stress than those who continued to smoke. These findings are consistent across women of different nationalities, as the studies were conducted in different countries. The findings confirm the relationship between stress and smoking during pregnancy and are consistent with previous reports [50,51]. Furthermore, stress has also been linked to smoking in the general population [52]. The belief that smoking is an effective strategy for coping with stress in pregnancy may lead women to continue smoking despite the pressure they receive from the people around them and knowledge about the risks to their health and that of their baby [6].

The third objective was to determine whether there is a relationship between stress and postpartum smoking relapse. Of the 15 studies reviewed, only three analysed the relationship between these two variables [28,32,39] and found it to be statistically significant. That is, women who experience higher levels of stress are more likely to relapse. Previous studies (e.g., [53]) reported similar results, finding that stress is a risk factor for postpartum smoking relapse. Furthermore, in a qualitative study in which women were asked why they relapsed, stress was the most frequently cited variable as well as a lack of coping skills [54]. Thus, if women who have quit smoking have not learned coping strategies to deal with stress after childbirth, they may turn to smoking to relieve their symptoms in times of high stress, and the behaviour will be negatively reinforced. It is, therefore, imperative that smoking cessation interventions during this period include stress management as part of the treatment.

The final objective was to examine the direction of the relationship between stress and perinatal smoking, depending on whether these were considered the dependent or independent variables in the studies. This approach enabled us to determine whether stress is a cause or a consequence of smoking. The results of the review show that there is a relationship between stress and smoking and that women smokers tend to have significantly higher levels of stress than non-smokers. However, the relationship between stress and smoking during pregnancy may be bidirectional: experiencing stress may lead pregnant women to smoke in order to manage the stress, or smoking may also lead to stress, generated by the awareness of the possible detrimental effects on the health of the woman and her baby.

Most studies (*n* = 13) included stress as the independent variable, with stress being considered a predictor of smoking during pregnancy [6,28,30,34,35,36,37,38,39,40,41] and in the postpartum period [28,32,39]. Eleven of these studies found that the relationship between stress and smoking in both pregnancy and the postpartum period was significant, and stress was thus considered the cause of or a risk factor for smoking. Specifically, women are more likely to smoke during pregnancy due to higher levels of stress [6,30,39,41], numerous stressful life events [28,35,36] and the presence of stress [40]. Conversely, women are more likely to quit smoking when they experience lower levels of stress [34,39]. Similarly, women are more likely to relapse postpartum when they experience numerous stressful life events [28,32] and higher levels of stress [29].

By contrast, the two studies addressing the relationship between smoking and stress [31,33], with stress considered the dependent variable, reported a statistically significant relationship, with smoking being a risk factor for stress during pregnancy.

Thus, the relationship may be bidirectional. Stress may lead women to smoke as a strategy to regulate the emotions and sensations associated with stress, which is thus a predictor of perinatal smoking. On the other hand, smoking may also generate stress, as it has been found that nicotine may exacerbate stress symptoms due to dependence and withdrawal symptoms [55]. Furthermore, smoking during pregnancy may also create stress in women, owing to awareness about the negative effects on their own and their baby’s health and also to the social stigma regarding smoking during pregnancy [56]. In fact, concealment of tobacco consumption is very common in pregnant women [57]. However, most of the research reviewed here considered stress to be the risk factor or cause of smoking in women during pregnancy and postpartum. Previous reviews conducted with other populations of women (non-pregnant) have found results in line with the findings found in the present study [58,59].

This is the first review to examine whether there is a relationship between stress and smoking in pregnancy and postpartum and the direction of this relationship. However, several limitations can be highlighted. First, the absence of a unified definition of the term stress is an important limitation. For example, some authors consider stressful life events as indicators of stress [25,26,27,28,29,30,31,32,33,34,35,36,37], others consider perceived stress [6,29,30,31,33,41], and others [38] consider adverse psychosocial circumstances. In addition, different parameters were assessed depending on the objectives of each study: level of stress [6,30,39,41] or number of stressful life events and their severity [28,32,35,36,37]. The different approaches also affect the choice of instrument used, thus hampering comparison of the results of the different studies.

On the other hand, most studies used self-report questionnaires to assess tobacco consumption. However, in this population, it is important to verify self-reports of abstinence with biochemical tests, as there may be a high rate of concealment of tobacco use due to a social desirability effect because consuming tobacco during pregnancy is viewed unfavourably by society [57]. In this review, only six of the fifteen studies used biochemical tests to verify the participants’ self-report of abstinence, such as measurement of expired air carbon monoxide [29] and tests for cotinine in saliva [29,41] and urine [6,34,35,60].

Other limitations included the small size of some samples [30] and the type of samples selected, e.g., low-income women [30,35], making it difficult to generalise the results to the rest of the population of pregnant women who smoke. Likewise, none of the studies analysed came from countries in Africa, Asia or Australia. Research is needed in these countries in order to analyse possible cultural differences.

The implications for clinical practice emerging from this review for consideration by intervention developers are that stress management should be a component of any smoking cessation intervention during pregnancy but not only in developed countries, as perinatal mental health is beginning to be taken into account in low- and middle-income countries as well [60]. This is a step forward in the development of effective interventions for smoking cessation during pregnancy in countries around the world because, as discussed throughout this paper, addressing psychological variables is essential.

### Future Directions

Future research could benefit from focusing on and addressing the findings reported above. Longitudinal studies that follow women from pregnancy to the postpartum period could provide valuable information. This would allow for a better understanding of how stress and smoking interact over time and how these factors affect maternal well-being and infant development. It would also be of great interest to implement strategies to identify women who are at risk for stress and smoking during pregnancy by assessing and monitoring these variables during prenatal visits. More studies are needed to understand the role of stress in perinatal smoking with a view to addressing this factor in smoking cessation interventions for pregnant women. It would also be interesting to investigate this relationship with other consumption modalities, such as the use of electronic cigarettes, given the increase in this practice in recent years.

## 5. Conclusions

The present review found that the most used instrument to assess the level of stress during pregnancy and the postpartum period is the PSS-14. It was also found that stress is related to smoking during the perinatal period as well as to smoking or cessation during pregnancy and to relapse in the postpartum period. Regarding the direction of the relationship, most of the findings indicate that stress is one of the causes of smoking. The findings have highlighted the need to improve smoking cessation interventions during pregnancy by incorporating stress management, as treating tobacco consumption is as important as treating the associated risk factors.

## Figures and Tables

**Table 1:** Instruments used in the reviewed studies to assess stress.

		Study														
		Businelle et al. (2013) [29]	Silveira et al. (2013) [33]	Beijers et al. (2014) [37]	White et al. (2014) [34]	Gilbert et al. (2015) [39]	Coleman-Cowger et al. (2016) [30]	Rockhill et al. (2016) [32]	Širvinskienė et al. (2016) [40]	Míguez and Pereira (2018) [6]	Yang et al. (2017) [35]	Allen et al. (2019) [28]	Crone et al. (2019) [38]	Fujita et al. (2021) [41]	Dhaliwal et al. (2022) [31]	Yakubu et al. (2023) [36]
Instruments	Perceived Stress Scale (PSS-14)															
	Life Events Inventory (LEI)															
	Ad hoc question on stress in pregnancy															
	Ad hoc questionnaire on stressful or traumatic experiences															
	Revised Prenatal Distress Questionnaire (NuPDQ)															
	Cambridge Worry Scale (CWS)															
	Everyday Stressors Index (ESI)															
	Stressful Life Events of PRAMS (PRAMS SLE)															
	Event questionnaire															

**Table 2:** Relationship between stress and smoking and/or smoking cessation during pregnancy.

Study	Objective	Sample	Stress Assessment	Results	Conclusions
* Silveira et al. (2013) [33] USA	To assess the correlates of high levels of perceived stress in a group of Hispanic women with high levels of stress during pregnancy.	979 Hispanic pregnant women screened at 12, 21 and 30 weeks of pregnancy.	Perceived Stress Scale (PSS-14)	The relationship between stress and smoking was significant, with a greater number of cigarettes associated with higher stress scores (OR = 2.2; *p* < 0.05).	Tobacco consumption increases the likelihood that women will experience stress during pregnancy.
Beijers et al. (2014) [37] The Netherlands	To examine whether the severity of different types of EVD is associated with continued smoking during mid-pregnancy.	2287 pregnant women (14–19 weeks) - Continued smoking: 113 - Quit smoking: 290 - Non-smokers: 1883	Event questionnaire	Perceived severity of SLE was not significantly associated with continued smoking in pregnant women (*p* > 0.05).	There is an association between SLE and smoking during pregnancy. However, smoking was not associated with the severity of SLE.
* White et al. (2014) [34] USA	To investigate possible predictors of spontaneous quitting among pregnant smokers, including stress.	349 pregnant women (>25 weeks) - Quit smoking: 118 - Continued smoking: 231	Not stated	Mean stress scores: - Quit smoking: 4.7 - Continued smoking: 5.5 (*p* < 0.01)	Women who quit smoking reported lower average levels of stress than women who continued to smoke.
* Gilbert et al. (2015) [39] Canada	To analyse the rates and determinants of smoking cessation during pregnancy.	1586 women who had a baby and were smoking before pregnancy. Subjects were interviewed between 5 and 15 months after delivery.	Ad hoc question on stress in the 12 months prior to delivery	Stress before or during pregnancy was associated with higher odds of not quitting smoking during pregnancy (OR = 1.39).	Stress reduces the likelihood that women will quit smoking during pregnancy.
* Coleman-Cowger et al. (2016) [30] USA	To identify differences between pregnant women who currently smoke and those who quit smoking 90 days before their first antenatal visit.	130 pregnant women (1st or 2nd trimester)	Perceived Stress Scale (PSS-14)	Women who smoked during pregnancy had higher mean stress scores than those who quit (*p* < 0.05).	Higher stress increases the likelihood of continued smoking, and lower stress increases the likelihood of quitting.
* Širvinskienė et al. (2016) [40] Lithuania	To investigate psychosocial predictors of smoking during pregnancy, including stress and distress.	514 mothers assessed between the second and third day after delivery.	Ad hoc questions about emotional stress during pregnancy.	Percentage of women with stress: - Smokers: 19.4% - Non-smokers: 13.8% (*p* > 0.05)	Stressful experiences were not significantly related to smoking during pregnancy.
* Yang et al. (2017) [35] USA	To assess the role of chronic stress levels in explaining the relationship between socioeconomic status and persistent prenatal smoking.	370 pregnant women evaluated at 5–13, 14–26 and 27–36 weeks gestation. - 84 smokers - 202 non-smokers - 84 quit spontaneously	Everyday Stressors Index (ESI)	Average chronic stress scores: - Smokers: 33.5 - Non-smokers: 27.8 - Spontaneous quitters: 33.5 (*p* < 0.001)	Mean scores on chronic stress are higher in the group of smokers than in non-smokers and those who quit smoking spontaneously. Stress during pregnancy is significantly related to smoking.
* Míguez and Pereira (2018) [6] Spain	To assess the prevalence of smoking in the first trimester of pregnancy and associated variables.	760 pregnant women (<20 weeks)	Perceived Stress Scale (PSS-14)	Mean scores on the PSS-14: - Smokers: 21.26 - Non-smokers: 16.24 (*p* < 0.001).	Women who smoked had higher mean levels of stress than non-smokers. Stress predicts smoking during pregnancy.
* Allen et al. (2019) [28] USA	To examine the association between SVEs in the year prior to delivery and perinatal smoking.	15,136 pregnant women - 7308 smokers - 7828 non-smokers	Life Events Inventory (LEI)	Mean stress scores: - Smokers: 3.29 - Non-smokers: 2.53 (*p* < 0.001)	Stress significantly increases the likelihood of women smoking during pregnancy.
Crone et al. (2019) [38] The Netherlands	To identify different groups of pregnant women according to their behavioural, psychosocial and socio-economic characteristics in health behaviour during pregnancy.	2455 women (12 weeks) - 3.8% continued smoking - 10.2% quit spontaneously - 86% never smoked during pregnancy	Revised Prenatal Distress Questionnaire (NuPDQ)Cambridge Worry Scale (CWS)	Mean NuPDQ scores: - Non-smokers: 1.8 - Quit: 4 - Smokers: 11.8Mean CWS scores: - Non-smokers: 1.9 - Quit: 3.6 - Smokers: 6.5	There is a link between stress and smoking during pregnancy.
* Fujita et al. (2021) [41] Brazil	To investigate how social and psychological characteristics differ between women who smoke and women who do not smoke during pregnancy.	269 pregnant women (< 24 weeks) - Smokers: 94 - Non-smokers: 175	Perceived Stress Scale (PSS-14)	Mean scores on the PSS-14: - Smokers: 24.7 - Non-smokers: 18.5 (OR = 1.07; *p* < 0.001).	Women who continued to smoke during pregnancy have higher mean scores on perceived stress than non-smokers.
* Dhaliwal (2022) [31] USA	To characterise maternal psychosocial stress to identify socio-demographic, biological, behavioural and health correlates of stress domains (overwhelm, anhedonia and lack of control).	1079 pregnant women (17–27 weeks)	Perceived Stress Scale (PSS-14)	Smoking during pregnancy was associated with the domain of lack of stress control (*p* < 0.005).	Analysis of the domains of stress and its relationship to smoking in pregnancy provides some insight into which specific stress symptoms are related to smoking in pregnant women.
* Yakubu et al. (2023) [36] USA	To examine smoking patterns in pregnant women who experienced SLE.	24,209 pregnant women (3rd trimester) - 1841 smokers - 21,990 non-smokers	Stressful Life Events of PRAMS (PRAMS SLE)	Of the group of women with the highest stress: - Smokers: 9.7% - Non-smokers: 1.6% (*p* < 0.001)	Women who reported higher stress were more likely to smoke during pregnancy.

*: statistically significant difference; SLE: stressful life events.

**Table 3:** Relationship between stress and postpartum relapse.

**Study**	**Objective**	**Sample**	**Stress Assessment**	**Results**	**Conclusions**
* Businelle et al. (2013) [29] USA	To examine, using different models, the multiple mechanisms linking socio-economic status to postpartum smoking relapse.	251 postpartum women who quit smoking before or during pregnancy. Subjects were divided into two groups: - Relapsed - Remained abstinent	Perceived Stress Scale (PSS-14)	The correlation between the PSS-14 scores and relapse was 0.145 (*p* < 0.05). The correlation between PSS-14 scores and craving: - Urge to smoke: 0.203 - Thinking about smoking: 0.306 - Desire to smoke: 0.196 (*p* < 0.001)	Stress was significantly related to smoking relapse after 8 weeks postpartum, as women who had relapsed reported higher levels of stress. In addition, stress was significantly related to different symptoms of craving.
* Rockhill et al. (2016) [32] USA	Identify characteristics associated with postpartum smoking relapse.	13,076 women	Stressful Life Events of PRAMS (PRAMS SLE)	Percentage of women who relapsed: -With 1–2 stressors: 37.9% -With more than 6 stressors: 49.7% (*p* < 0.001)	The percentage of women who relapsed increased significantly with the number of stressors they had experienced.
* Allen et al. (2019) [28] USA	To examine the association between SLEs and postpartum smoking relapse.	7151 women - 31,226 relapsed - 4025 remained abstinent	Life Events Inventory (LEI)	Average SLE scores - Relapsed: 2.71 - Abstinent: 2.35 (*p* < 0.001)	Mean SLE scores were significantly higher in the group of women who relapsed postpartum.

*: statistically significant difference; SLE: stressful life events.

## Supplementary Materials

The following supporting information can be downloaded at: https://www.mdpi.com/article/10.3390/brainsci15010013/s1, Table S1: MMAT: Quantitative Descriptive Studies.
