# Supplementary material for: The Connection Between Stress and Women’s Smoking During the Perinatal Period: A Systematic Review
Source: Brain Sci. 2024 Dec 26;15(1):13. doi: 10.3390/brainsci15010013 (PMC11764003; doi:10.3390/brainsci15010013)
Supplement: Supplementary file 1 [file brainsci-15-00013-s001.zip › brainsci-3358759-supplementary.pdf]

MMAT: QUANTITATIVE DESCRIPTIVE STUDIES

STUDY	S1. Are there clear research questions?	S2. Do the collected data allow to address the research questions?	4.1. Is the sampling strategy relevant to address the research question?	4.2. Is the sample representative of the target population?	4.3. Are the measurements appropriate?	4.4. Is the risk of nonresponse bias low?	4.5. Is the statistical analysis appropriate to answer the research question?
**Businelle et al. (2013)**	Yes	Yes	Yes	Yes	Yes	?	Yes
**Silveira et al. (2013)**	Yes	Yes	Yes	No	Yes	?	Yes
**Beijers et al. (2014)**	Yes	Yes	Yes	Yes	Yes	Yes	Yes
**White et al. (2014)**	Yes	Yes	Yes	Yes	Yes	?	Yes
**Gilbert et al. (2015)**	Yes	Yes	Yes	Yes	Yes	?	Yes
**Coleman-Cowger (2016)**	Yes	Yes	Yes	Yes	Yes	?	Yes
**Rockhill et al. (2016)**	Yes	Yes	Yes	Yes	Yes	Yes	Yes
**Širvinskienė et al. (2016)**	Yes	Yes	Yes	Yes	Yes	?	Yes
**Míguez and Pereira (2018)**	Yes	Yes	Yes	Yes	Yes	?	Yes
**Yang et al. (2017)**	Yes	Yes	Yes	Yes	Yes	Yes	Yes
**Allen et al. (2019)**	Yes	Yes	Yes	Yes	Yes	Yes	Yes
**Crone et al. (2019)**	Yes	Yes	Yes	Yes	Yes	?	Yes
**Fujita et al. (2021)**	Yes	Yes	Yes	Yes	Yes	?	Yes
**Dhaliwal et al. (2022)**	Yes	Yes	Yes	Yes	Yes	?	Yes
**Yakubu et al. (2023)**	Yes	Yes	Yes	Yes	Yes	?	Yes

Note. ?: Cannot tell

Contributors: Yara Quiero and Cristina M. Posse

Hong, Q.N.; Pluye, P.; Fàbregues, S.; Bartlett, G.; Boardman, F.; Cargo, M.; Dagenais, P.; Gagnon, M.P.; Griffiths, F.; Nicolau, B.; et al. Mixed methods appraisal tool (MMAT). Version 2018. McGill University, 2018, http://mixedmethodsappraisaltoolpublic.pbworks.com/w/file/fetch/127916259/MMAT_2018_criteria-manual_2018-08-01_ENG.pdf
